# *Cutibacterium acnes* as a cause of late prosthetic valve endocarditis: a case report

**DOI:** 10.1093/ehjcr/ytag214

**Published:** 2026-03-18

**Authors:** Amir Hakanovic, Filipe Patricio, Andres Spirig, Michel Zuber, Tobias A Fuchs

**Affiliations:** Department of Cardiology, Kantonsspital Aarau, Tellstrasse 25, 5001 Aarau, Switzerland; Department of Cardiology, Kantonsspital Aarau, Tellstrasse 25, 5001 Aarau, Switzerland; Department of Radiology, Kantonsspital Aarau, Tellstrasse 25, 5001 Aarau, Switzerland; Department of Cardiology, Kantonsspital Aarau, Tellstrasse 25, 5001 Aarau, Switzerland; Department of Cardiology, Kantonsspital Aarau, Tellstrasse 25, 5001 Aarau, Switzerland

**Keywords:** Aortic disease, *Cutibacterium acnes*, Pseudoaneurysm, Mechanical valve prosthesis, Infective endocarditis, Case report

## Abstract

**Background:**

Dehiscence of a composite graft following aortic valve (AV) and ascending aorta replacement represents a rare but potentially life-threatening complication. The need for re-operation after a Bentall procedure is usually related to the development of an endocarditis, formation of (pseudo)aneurysms, or recurrent dissection. We report a rare case of late pseudoaneurysm formation due to *Cutibacterium acnes* (*C. acnes*) endocarditis.

**Case summary:**

A 52-year-old man was admitted to the emergency department due to dizziness, diplopia, right-sided hemiparesis, and dysarthria. Five and a half years ago, the patient underwent a replacement of the AV and entire ascending aorta due to a bicuspid AV with combined valvular disease and ascending aortic aneurysm. Transthoracic echocardiography revealed a large, perfused posterior pseudoaneurysm caused by the dehiscence of the composite graft, while the mechanical valve remained well functioning. After 8 days of incubation, *C. acnes* was detected in blood cultures. The patient underwent re-operation, including replacement of the composite graft, reimplantation of the coronary ostia, and replacement of the hemiarch. The post-operative course was uneventful.

**Discussion:**

The dehiscence of a composite graft is a rare but serious complication following AV and aortic replacement with a high mortality. *Cutibacterium acnes* is a slow-growing, biofilm-forming bacterium, considered a typical pathogen in infective endocarditis (IE) in the presence of prosthetic material. Cardiac imaging is the cornerstone of IE diagnostic process, often requiring multimodality imaging approach. The management of *C. acnes* endocarditis typically involves a combination of antibiotic therapy and frequently surgical intervention.

Learning points

- A pseudoaneurysm may develop years after surgical aortic replacement.
- *Cutibacterium acnes* is a slow-growing and low-virulence bacterium that requires prolonged antibiotic therapy.
- In addition to advanced echocardiography, multimodality imaging plays a key role in the diagnosis of infective endocarditis and its complications.

## Introduction

Dehiscence of a composite graft following aortic valve and ascending aorta replacement is a rare but potentially life-threatening complication.^1^ In this report, we describe a patient who developed a pseudoaneurysm due to *Cutibacterium acnes* (*C. acnes*) endocarditis, 5.5 years after the initial operation.

*Cutibacterium acnes* is a slow-growing, gram-positive anaerobe bacterium that belongs to the normal skin flora and has the ability to form biofilms. Over recent years, it has become increasingly recognized as a clinically relevant microorganism in delayed post-surgical infections, particularly in the context of cardiac surgery and prosthetic material.^2^

One of the challenges in diagnosing *C. acnes* endocarditis lies in the subtle or even absent clinical signs of systemic inflammation. Its low virulence, biofilm production, and slow growth can allow the infection to persist silently over extended periods—sometimes months or even years—before clinical symptoms emerge.^3^

Treatment generally requires prolonged antibiotic therapy. *Cutibacterium acnes* typically shows susceptibility to beta-lactam antibiotics such as penicillins and cephalosporins. However, in cases with complications like graft dehiscence or pseudoaneurysm formation, surgical intervention is frequently necessary.^4,5^

## Summary figure

**Table:** 

5.5 years prior	Symptomatic severe aortic valve insufficiency with moderate stenosis due to a bicuspid valve. Severely dilated left ventricle (LV) with severely impaired left ventricle ejection fraction (20%). Ascending aortic aneurysm (60 mm) Total replacement of the ascending aorta and hemiarch, reimplantation of the coronary arteries in the Button technique, and replacement of the aortic valve with a Medtronic MHV mechanical prosthesis
Annual follow-up	First post-operative follow-up and subsequent annual evaluations showed good function of the mechanical aortic valve prosthesis, with an improved left ventricular ejection fraction of 36%
Day 0	Presentation with transient ischaemic attack (dizziness, diplopia, right-sided hemiparesis, and dysarthria)
Day 1	Fever (38°C), transthoracic and transoesophageal echocardiography revealed the dehiscence of the composite graft with a perfused subvalvular pseudoaneurysm. Blood cultures revealed *C. acnes* (time to positivity 8 days)
Day 19	Re-operation with debridement and mechanical composite graft reconstruction in the modified Bentall technique with a 25 mm valve prosthesis (Medtronic AVG conduit) and reimplantation of the coronary arteries. In addition, hemiarch replacement was carried out, and a single coronary artery bypass graft (CABG) was performed using the left internal mammary artery (LIMA) to the left anterior descending artery (LAD)

## Case presentation

A 52-year-old man presented to the emergency department with acute-onset dizziness, diplopia, right-sided hemiparesis, and dysarthria. His past medical history revealed a replacement of the aortic valve (AV) (Medtronic STD MITRAL, Model 500D) and entire ascending aorta (intervascular, bulged graft, 34 mm, Intergard Woven 28 mm) replacement using the button technique due to combined AV disease in the setting of a bicuspid AV and ascending aortic aneurysm. Two months prior, routine cardiologic examination had shown normal prosthetic valve function with mildly reduced left ventricular systolic function. On admission, the patient was hypertensive (147/88 mmHg), in normal sinus rhythm (80 bpm), and febrile (38°C). His skin was unremarkable. Neurological examination revealed right-sided hemiparesis and dysarthria. Laboratory work-up demonstrated elevated inflammatory markers.

**Figure 1:**
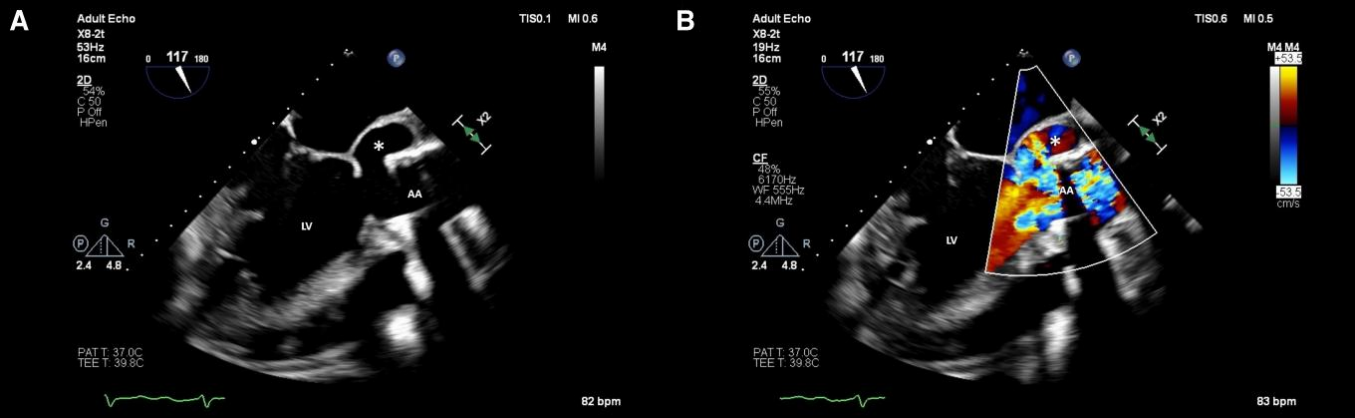
(*A*) Two-dimensional transoesophageal echocardiography demonstrating a dehiscence of the composite graft. (*B*) Colour Doppler imaging showing perfusion of the subvalvular pseudoaneurysm. LV, left ventricle; AA, ascending aorta; asterisk (*), pseudoaneurysm.

An urgent cranial computed tomography (CT) with extracranial computed tomography angiography (CTA) ruled out intracranial haemorrhage, perfusion deficit, and high-grade stenosis of the cerebral arteries. A followed transthoracic and transoesophageal echocardiography (TTE/TOE) revealed a large, perfused, posterior pseudoaneurysm with partial dehiscence of the composite graft. The left ventricle was severely dilated with moderately reduced systolic function (biplane ejection fraction 36%). No oscillating structures or thrombotic deposits were detected (*Figure 1*/see Supplementary material online, *Video S1*).

In summary, the patient met one major modified Duke criterion (dehiscence of the composite graft) and four minor criteria [fever, positive rheumatoid factor, transient ischaemic attack, and a predisposing cardiac condition (prosthetic graft)], fulfilling the definition of definitive prosthetic valve endocarditis. After obtaining blood cultures, empiric intravenous antibiotic therapy with amoxicillin/clavulanic acid (6 × 2.2 g/day) was initiated.

**Figure 2:**
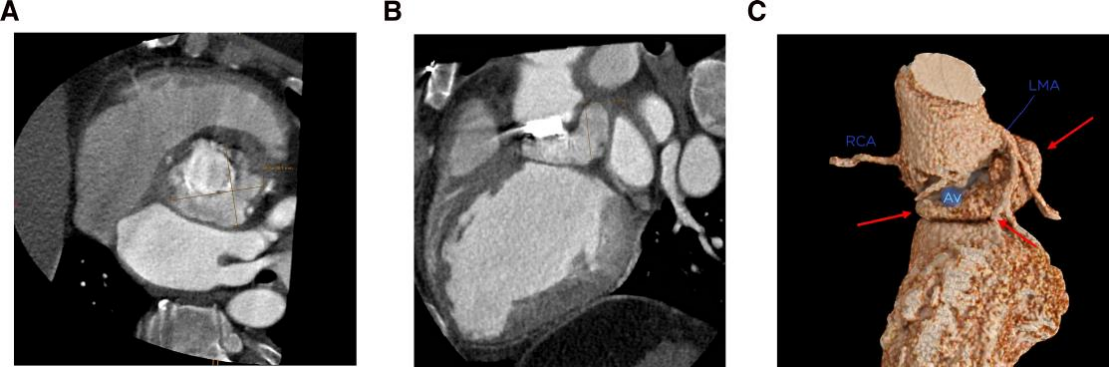
Multiplanar reconstructions (*A*, axial; *B*, coronal) and 3D rendering (*C*) of electrocardiogram -gated computed tomography angiography of the heart and the ascending aorta showing a subvalvular, perfused pseudoaneurysm (arrows) measuring 5.9 × 4.8 × 3.3 cm. AV, aortic valve; RCA, right coronary artery; LMA, left main artery.

Following a multidisciplinary endocarditis team discussion, surgical intervention was recommended. Preoperative work-up included CT imaging of the coronary arteries and thoracic aorta, confirming dehiscence of the mechanical composite graft with formation of a paravalvular pseudoaneurysm and suspected periprosthetic abscess formation along the ascending aortic graft (*Figure 2*).

Invasive coronary angiography revealed a significant stenosis in the mid-LAD.

In the further course, the patient developed acute cholecystitis, which was treated conservatively and led to a delay of the planned surgery. After 8 days, blood cultures showed growth of *C. acnes*, and the antibiotic therapy was adjusted according to the resistance profile to ceftriaxone 2 g/24 h.

**Figure 3:**
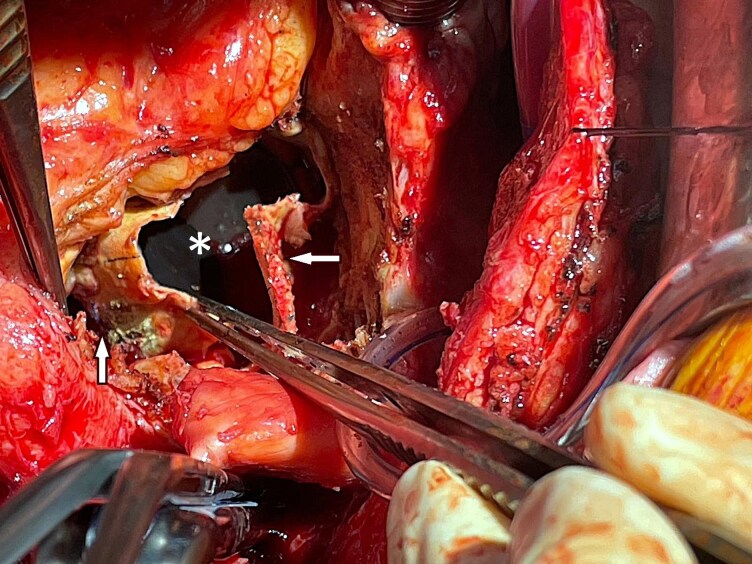
Intraoperative view showing the mechanical aortic valve prosthesis (*), the ascending aortic graft (left arrow), and the pseudoaneurysm cavity after surgical debridement (right arrow).

Finally, a re-operation was performed, which included debridement and mechanical composite graft reconstruction using the modified Bentall technique with a 25 mm valve prosthesis and reimplantation of the coronary buttons (Medtronic AVG conduit). Hemiarch replacement was carried out under deep hypothermic circulatory arrest (DHCA) with a 28 mm sidearm graft (Gelweave). A single CABG was performed using the left internal mammary artery to the LAD (*Figure 3*). The patient was placed on long-term antibiotics, made an uneventful recovery, and was subsequently discharged to cardiac rehabilitation.

## Discussion

Prosthetic valve endocarditis after a Bentall procedure is a severe complication that should be suspected in the presence of paravalvular abnormalities, as separation of the prosthetic valve from the conduit is often not feasible. Periannular involvement and paravalvular leakage are common findings in prosthetic valve endocarditis.^6^

Dehiscence of a composite graft is a rare but serious complication (occurring in 1%–3% of cases) following AV and aortic replacement, and it is associated with a high mortality rate.^1,6^

In this case, the infection with the slow-growing *C. acnes* (which has a median time-to-positivity of around 7 days, prompting some experts to recommend extended incubation for blood cultures) led to the formation of a pseudoaneurysm.^7^

*Cutibacterium acnes* is part of the skin’s normal flora, and it is a slow-growing bacterium capable of forming biofilms. Saha *et al*.^8^ reported that *C. acnes* is responsible for 3.1% of endocarditis cases, making it comparable in prevalence to the HACEK group. Recently *C. acnes* has been considered as a typical microorganism in infective endocarditis (IE) in the presence of intracardiac prosthetic material.^9^ Since patients often present without the typical signs of inflammation, diagnosing *C. acnes* endocarditis can be particularly challenging.^7^

The diagnosis of endocarditis is made according to the modified Duke criteria. The first-line imaging modality is TTE. If TTE results are negative or non-diagnostic and clinical suspicion remains high, or if there is a prosthetic valve or cardiac implantable electronic device (CIED), TOE is recommended. If TOE is inconclusive or there is suspicion of paravalvular or periprosthetic complications, a cardiac CT scan is recommended.^10^

Transoesophageal echocardiography is more effective than CT in detecting valvular lesions, especially small vegetations (<10 mm) and valve perforations. Another advantage of TOE is that it does not expose the patient to radiation or contrast agents. However, in the case of prosthetic valves, the accuracy of TOE can be reduced due to the acoustic shadowing caused by the prosthetic material.

Computed tomography, on the other hand, has a high sensitivity for detecting paravalvular lesions and is superior to TOE when it comes to identifying complications such as abscesses, pseudoaneurysms, and fistulas. Computed tomography is also less invasive for the patient and provides a comprehensive view of extracardiac structures and coronary arteries, which is critical for surgical planning.^11,12^

In addition to echocardiography and cardiac CT, positron emission tomography/computed tomography (PET/CT) plays an important role in the diagnostic work-up of prosthetic valve endocarditis, particularly when echocardiographic findings are inconclusive. PET/CT provides incremental diagnostic value by detecting inflammatory activity at the prosthetic valve and periprosthetic tissues and may help reclassify cases categorized as ‘possible’ IE by the modified Duke criteria.^10^

The management of *C. acnes* endocarditis typically involves a combination of antibiotic therapy and surgical intervention when indicated. First-line antibiotics for *C. acnes* infections include penicillin, ceftriaxone, and rifampicin.^4,5^

The role of *C. acnes* in this case emphasizes the importance of extended blood culture incubation and thorough imaging when prosthetic endocarditis is suspected. Although *C. acnes* is less virulent than other pathogens, untreated infections can lead to severe complications, including abscesses or pseudoaneurysms. In this case, a surgical revision, involving radical debridement and reimplantation of the composite graft, was necessary for successful management.

## Conclusion

This case underscores several important aspects in prosthetic valve endocarditis. While late-onset *C. acnes* infection is uncommon, its potential for atypical presentations—including neurological symptoms—poses diagnostic challenges. The slow-growing, biofilm-forming nature of the pathogen highlights the need for prolonged blood culture incubation. A combination of multimodality imaging, microbiological diagnostics, and interdisciplinary therapy is crucial for successfully managing such complications. By sharing this case, we aim to reinforce clinical awareness, support early recognition, and provide practical insights for the management of similar patients with cardiovascular implants.

## Supplementary Material

ytag214_Supplementary_Data

## Data Availability

No new data were generated or analysed in support of this research.

## Lead author biography

Amir Hakanovic is a cardiology trainee at the Department of Cardiology, Kantonsspital Aarau, Switzerland. He is currently in the final year of his specialist training in cardiology. His main clinical and academic interests focus on heart failure, including the diagnosis and management of advanced heart failure syndromes.

## Notes

**Consent:** Written informed consent for publication has been obtained from the patient in accordance with the COPE best practice guidelines. The individual reported on is fully aware of the nature of the publication and of the possible consequences of the reporting. A signed consent form is available and can be provided upon request, in line with the journal’s consent policy.
